# Differences in Influence of Particle Size on the Adsorption Capacity between Deformed and Undeformed Coal

**DOI:** 10.1021/acsomega.0c06306

**Published:** 2021-02-12

**Authors:** Yanwei Liu, Jian Miao, Hongkai Han, Peng Xu

**Affiliations:** † School of Safety Science and Engineering, Henan Polytechnic University, Jiaozuo454003, China; ‡ State Key Laboratory Cultivation Base for Gas Geology and Gas Control (Henan Polytechnic University), Jiaozuo454003, China; § The Collaborative Innovation Center of Coal Safety Production of Henan Province, Jiaozuo454003, China; ∥ School of Intelligent Engineering, Shandong Management University, Jinan250357, China; ⊥ Key Laboratory of Public Safety Management Technology of scientific research and innovation platform in Shandong Universities during the 13th Five Year Plan Period, Shandong Management University, Jinan250357, China; # College of Science, China Jiliang University, Hangzhou310018, China

## Abstract

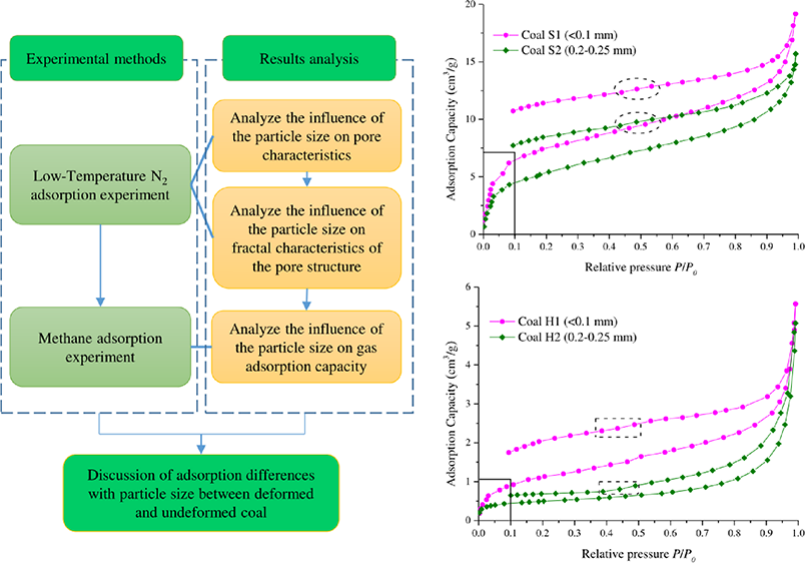

The prediction exactness of coalbed methane (CBM) content and productivity correlates closely with the gas adsorption rules of coal, but there is a noticeable difference in the gas adsorption rules between deformed and undeformed coal. One of the main factors affecting the gas adsorption capacity of coal is pore structure, which is affected by the particle size, and it is also one of the essential differences between deformed and undeformed coal. In this work, we experimentally study the law of the pore structure and gas adsorption capacity with the particle size. Results show that the specific surface area and the pore volume of undeformed coal increase significantly as the particle size decreases, while the variation trend of those of deformed coal is insignificant. The fractal dimension *D*_2_ and the particle size show a U-shaped correlation. The fractal dimension *D*_2_ reaches the minimum value at a coal particle size of 1–3 mm and 0.2–0.25 mm for deformed and undeformed coal, respectively. The *D*_2_ values of deformed and undeformed coal are closest in the case of particle sizes smaller than 0.1 mm. The difference in the adsorption capacity between deformed and undeformed coal diminishes with the decreasing particle size as the pore structure characteristics of undeformed coal gradually approach those of deformed coal. The obtained conclusions provide a theoretical foundation for the selection of the particle size of coal samples so as to predict coal and gas outburst disasters and CBM productivity accurately.

## 1 Introduction

The coal seam contains a large number of pores and fracture systems.^1,2^ Coal is a kind of a porous medium with a complex structure.^3^ In the study, undeformed coal (denoted as Coal H), whose overall structure is fairly intact, is not greatly affected by tectonic pressure.^4^ Compared to undeformed coal, deformed coal (denoted as Coal S) has experienced multiple geological tectonic stages and shown a low-strength and weakly cohesive form.^5^ Deformed coal has strong plasticity and ductile deformation, and its pore structure is different from that of undeformed coal. The reason is that due to one or more tectonic stresses, the pore structure of the deformed coal has severely changed, even affecting its adsorption capacity. In this study, deformed coal and undeformed coal are distinguished by their firmness coefficient (*f*): *f*>0.5 indicates undeformed coal, while *f*<0.5 indicates deformed coal.^5,6^ Currently, the gas outburst accident often occurs in the coal seam where deformed coal is developed. The difference in the adsorption capacity between deformed and undeformed coal is the key to predicting coal and gas outburst areas, while the particle size fairly affects the adsorption capacity of coal. There are certain requirements for the particle size when measuring the gas content and outburst prediction indicators. However, the relevant regulations on the particle size requirements for measuring work only rely on engineering experience and not on theoretical basis. Therefore, it is necessary to clarify influences of the particle size and tectonic deformation on the adsorption mechanism so as to further develop jobs of safe mining, CBM exploitation, and outburst prevention and control.

The gas adsorption capacity of coal is strongly correlated with its pore characteristics, so grasping the knowledge of pore structure is the foundation to understand the gas adsorption capacity of coal.^7,8^ The International Union of Pure and Applied Chemistry (IUPAC)^9^ divides pores into three groups, namely, micropores (smaller than 2 nm), mesopores (2–50 nm), and macropores (larger than 50 nm). Micropores and mesopores are generally perceived as adsorption pores, which primarily decide the coal adsorption capacity.^10^ Many scholars focused on deformed coal, while some studied the influence of particle size on pore characteristics and adsorption capacity. Yan et al.^11^ conducted research on the influence of tectonic deformation on the methane adsorption capacity of coal using a high-pressure volumetric method. The results illustrated that the adsorption capacity of deformed coal is a bit higher than that of undeformed coal. Also, Lu et al.^12^ demonstrated that the fractal dimension increases on enhancing the tectonic deformation. Li et al.^13^ proved that the specific surface area (SSA) and pore volume (PV) present a trend of “increase–decrease–increase” as the tectonic deformation enhances. Zou and Rezaee^14^ found that the adsorption capacity increases with the decreasing particle size using the high-pressure volumetric method. Liu et al.^15^ employed a low-temperature N_2_ adsorption method to estimate the SSA and PV of bituminous coal with medium and high ash contents. The results depict that SSA and PV increase with the growth of the particle size. Mastalerz et al.^16^ proposed analytical particle sizes, which can best represent the “real” value of coal and shale, 60 and 200 mesh, respectively. Hou et al.^17^ selected bituminous coal with different extents of tectonic deformation in different regions, and they found that the particle size has great effects on the mesopore structure but irregular effects on the micropore structure. Furthermore, the particle size effect on the pore characteristics has been weakened in deformed coal. However, we still lack knowledge of the particle size effect on the pore structure and adsorption capacities of typically deformed and undeformed coal (anthracite) in the same coal seam.

In recent years, researchers have adopted various methods to study the pore characteristics, including N_2_/CO_2_ adsorption,^1^ high-pressure mercury intrusion,^18,19^ small-angle X-ray scattering,^2^ small-angle neutron scattering,^20^ nuclear magnetic resonance,^21^ scanning electron microscopy, and transmission electron microscopy.^22^ Among these methods, the most frequently used method is low-temperature N_2_ adsorption, which turned to be a useful method for analyzing the pore structures of porous media in a previous related research study.^23^ Fractal dimension is a valid method to quantify the pore structure characteristics of coal. These characteristics are correlated with pore characteristic parameters, pore surface parameters, and adsorption capacity of coal.^24^ However, the fractal characteristics of different particle sizes of deformed and undeformed coal are still unclear.

As can be seen from the above review, some conclusions about influence laws of particle size on the adsorption capacity of coal have been made in previous studies.^3,11,14,17^ However, the difference in influence of particle size on adsorption capacity with between deformed and undeformed coal is still poorly studied. Researchers adopted the low-temperature N_2_ adsorption experiment and the Frenkel–Halsey–Hill (FHH) fractal model to analyze the pore structure of typically deformed and undeformed coal (anthracite) with different particle sizes. Moreover, we also analyzed the adsorption capacities of coal using the high-pressure volumetric method. It is worth noting that typical deformed and undeformed coal particles come from different working faces in the same coal mine. Three main purposes of this paper are as follows: (1) to investigate the difference in the variations of the pore structure and fractal characteristics between the deformed and undeformed coal with different particle sizes, (2) to study the gas adsorption capacity variations between the deformed and undeformed coal with particle size, especially the difference between them, and (3) to discuss the relationship among the tectonic deformation, particle size, and adsorption capacity from the perspective of mechanics.

## 2 Methodology

### 2.1 Sample and Preparation

**Figure 1:**
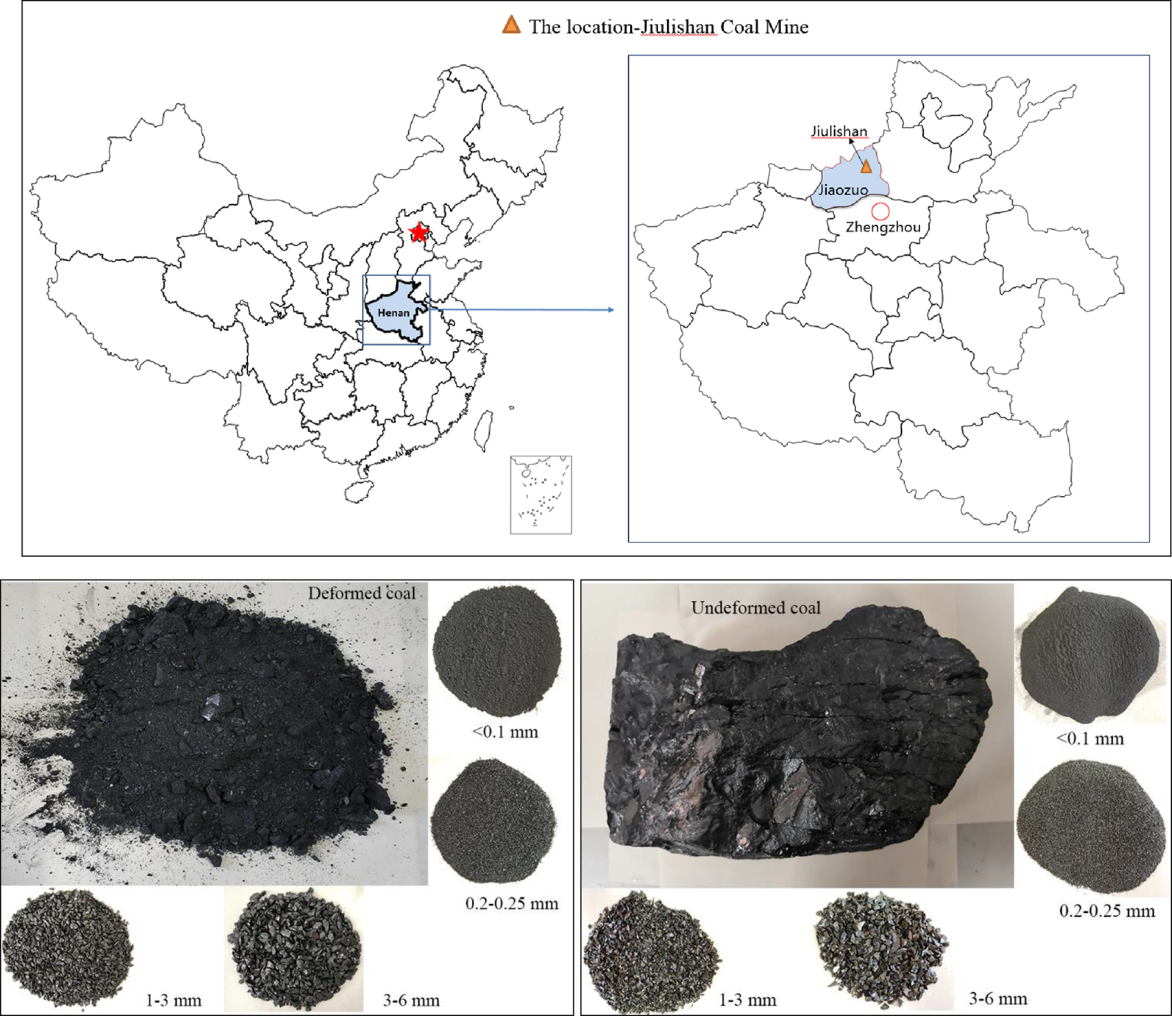
Distribution map and macroscopic features of deformed and undeformed coal by visual inspection.

When predicting coal and gas outburst disasters and coalbed methane (CBM) productivity, it is necessary to choose a representative coal sample with a specific size so as to determine the gas content of the coal seam, gas desorption index, and other parameters.^25,26^ We collected coal samples with different deformation degrees from the Jiulishan Coal Mine, which is located in the middle of the Jiaozuo Mining Area, Henan Province, China. This coal mine suffers from serious coal and gas outburst. The coal-bearing strata uplift along with the Taihang Mountains, forming NE-NNE-oriented faults and folds. They are mainly characterized by tectonic compression and shearing that make the coal seam undergo ductile shearing along the coal seam and result in different degrees of damage to the top and bottom of the coal seam, while the middle part of the coal seam is outside the bedding shear zone, which is slightly damaged. The undeformed coal samples (Coal H) were picked from the slightly damaged coal seam. These samples are integrated and blocky with obviously bedded structures, and they could not be broken by hand. The deformed coal samples (Coal S) were collected from the severely damaged coal seam. The coal body of the samples is crumpled, which could be easily broken into a powdery or granular shape by hand. The sample morphology is shown in Figure 1.

In light of the China standard GB/T 23561.12–2010, the hardiness coefficients of the two samples were measured (Table 1). We crushed the two coal samples mechanically and reduced them using a 6 mm standard sieve. In light of the China standard GB 474–2008, we used the coning and quartering method for coal sample division. Each sample was equipartitioned using the mixing and reduction method. Then, it was further sieved into four particle size fractions, namely, 3 to 6 mm, 1 to 3 mm, 0.2 to 0.25 mm, and less than 0.1 mm (Figure 1). Next, we conducted the proximate analysis, low-temperature N_2_ adsorption experiment, and methane adsorption experiment (high-pressure volumetric method) on each particle size fraction. The proximate parameters of each analyzed fraction obtained by air drying are listed in Table 1.

**Table 1:** Proximate Analysis Parameters of Two Kinds of Coal samples^a^

			proximate analysis (%)		
sample no.	particle size (mm)	hardiness coefficient	*M*_ad_	*A*_ad_	*V*_ad_
S1	<0.1	0.25	1.88	11.34	10.36
S2	0.20 to 0.25		1.75	10.57	9.38
S3	1 to 3		1.83	9.62	9.35
S4	3 to 6		1.86	10.67	9.82
H1	<0.1	1.51	1.68	7.50	9.65
H2	0.20 to 0.25		1.74	6.34	9.47
H3	1 to 3		1.72	5.93	9.43
H4	3 to 6		1.79	5.86	9.18

a Note: *M*_ad_ denotes the moisture content; *A*_ad_ is the ash yield; and *V*_ad_ represents the volatile matter.

### 2.2 Low-Temperature N_2_ Adsorption Experiment

An automatic surface area and pore analyzer (V-sorb 2800TP, Gold APP Instruments Corp., China) was used to determine the pore structures. Before carrying out the analysis, we first degassed the samples used for the adsorption analysis under vacuum conditions at 120 °C for 8 h, which aims to get rid of the adsorbed volatile substances. As for the N_2_ (77.35 K) adsorption measurements, we obtained both the adsorption and desorption isotherms in a relative pressure range of 0.01 to 0.995. Then, according to the Brunauer–Emmett–Teller (BET), Dubinin–Radushkevich (D-R), and Barrett–Joyner–Halenda (BJH) methods, the pore structural parameters were automatically calculated by computer software. Using the multipoint BET method, the BET SSA of each sample was obtained. By adopting the D-R method, we calculated the micropore volume and SSA. Meanwhile, the BJH SSA, PV, and pore size distribution (PSD) of pores with diameters ranging from 2 to 300 nm were determined using the aforementioned BJH method.

### 2.3 Methane Adsorption Experiment

In light of the China national standard GB/T 19560–2008, the methane adsorption experiment on samples was conducted by adopting the high-pressure volumetric method. An adsorption constant tester (WY-98B) manufactured at the Shenyang Branch of the China Coal Research Institute was used in the experiment. The source of the samples for the methane adsorption experiment was the same as that for the low-temperature N_2_ adsorption experiment. Each coal sample weighing 20–30 g was placed into a coal sample tank for adsorption isotherm testing. First, the coal sample was degassed for 4 h. Then, we inflated the coal sample tank using a methane inflatable bottle. The equilibrium time for gas filling was 7 h or 4 h. The pressure of the methane inflatable bottle was set to 1.6, 2.2, 3.0, 3.4, 4.2, 5.0, and 5.8 MPa. The adsorption equilibrium temperature was 30 ± 1 °C.

## 3 Results and Discussion

The pore structure of the coal body indicates the size, morphology, development degree, and mutual combination of pores contained in coal reservoirs. These characteristics are mainly reflected by the pore morphology, SSA, fractal dimension, and PV.

### 3.1 Differences in Influence of the Particle Size on Pore Characteristics between Undeformed and Deformed Coal

By conducting low-temperature N_2_ adsorption experiments, we obtained the pore characteristics of the deformed and undeformed coal samples with different particle sizes.

#### 3.1.1 N_2_ Adsorption/Desorption Isotherms

**Figure 2:**
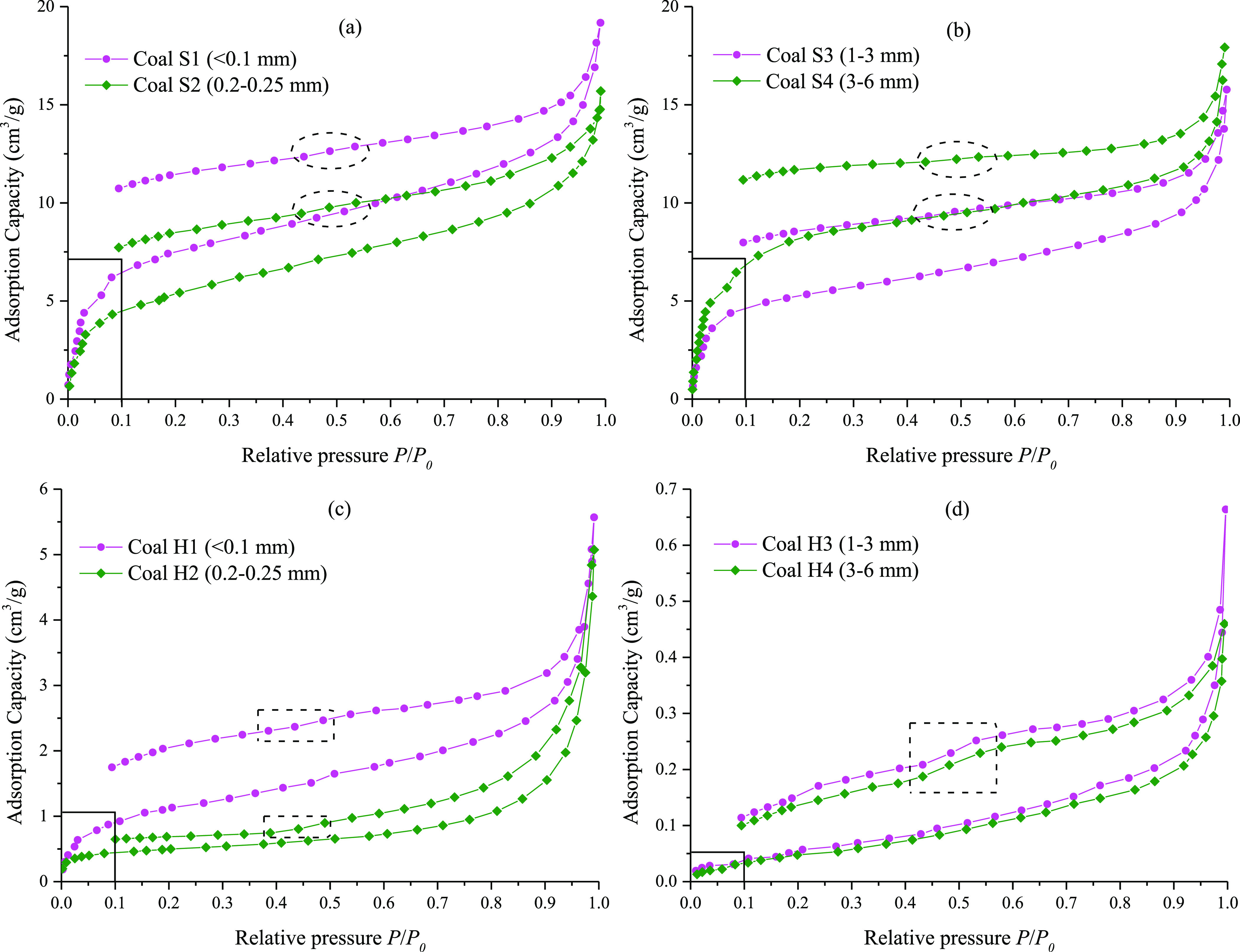
Low-temperature N_2_ adsorption isotherms of the coal samples. Note: (a) and (b) represent Coal S and (c) and (d) represent Coal H. *P* = equilibrium pressure and *P*_0_ = saturated vapor pressure.

The isotherms can be widely employed to characterize the pore shapes and sizes of the coal samples and calculate the parameters of various pore structures.^23^ The N_2_ adsorption and desorption isotherms of the coal samples examined in this study are shown in Figure 2, covering four particle size fractions. The maximum adsorption volume of Coal S ranges from 14.89 to 19.18 cm^3^/g. Its variation trend with the particle size is not obvious. In contrast, the maximum adsorption volume of Coal H ranges from 0.46 to 5.7 cm^3^/g, and the sample with smaller particle sizes adsorbs more nitrogen molecules. Deformed coal having a significantly stronger adsorption volume than undeformed coal demonstrates that the tectonic deformation can increase the PV. The reason is as follows: deformed coal has undergone long-term tectonic stress, and its strength is far greater than the mechanical broken stress in the laboratory. In other words, mechanical crushing in the laboratory is secondary damage to the coal sample, and long-term tectonic stress has previously destroyed it to an extremely tiny particle size. Therefore, the particle size of Coal S is already very small, and its adsorption capacity is close to the maximum value. The maximum adsorption capacity of Coal H increases significantly with the decrease of particle size, indicating that mechanical pulverization has a positive effect on the pore structure of Coal H, resulting in the adsorption capacity of Coal H gradually approaching that of Coal S.

The hysteresis loop type of the coal samples is similar to the characteristics of Type H1 recommended by IUPAC,^9,23^ where the adsorption isotherms increase gradually when *P*/*P*_0_ <0.8 and then increase rapidly when *P*/*P*_0_ approaches 1.0. When *P*/*P*_0_ <0.4, the hysteresis loop lacks closure, which is attributed to the swelling (chemical adsorption or structural deformation), nonreversible adsorption of micropores or incomplete equilibration during measurements.^27,28^ As shown in Figure 2(c,d), the adsorption capacity of Coal H with a smaller particle size is greater than that with a larger particle size at different *P*/*P*_0_ values, while Figure 2(a,b) shows that the adsorption capacity of Coal S with different particle sizes is relatively similar. The adsorption capacity of Coal S with four different particle sizes is relatively similar because deformed coal has undergone long-term tectonic stress, whose strength is far greater than the mechanical broken stress in the laboratory. It should be pointed out that in Figure 2(b), the adsorption capacity of Coal S4 (3–6 mm) is larger than that of Coal S3 (1–3 mm) and not the opposite. The reason is that when the particle size of Coal S is larger than the particle size limit, the initial velocity of gas desorption and diffusion reaches the maximum, and the adsorption capacity tends to be stable, almost no longer changing with the particle size.^29,30^ When *P/P_0_* is between 0.45 and 1, both Coal S and Coal H exhibit a distinct hysteresis loop, which shows breathable pores with two sides open and fine bottleneck pores with one side open. An inconspicuous inflection point appears when *P*/*P*_0_ = 0.5 on the desorption curves of Coal S, and it shows no variation with the particle size, which indicates that the ink-bottle pores barely exist in Coal S. A sudden drop on the isotherm of Coal H appears when *P*/*P*_0_ = 0.5. However, the drop gradually becomes inconspicuous as the particle size becomes smaller, which indicates that the quantity of ink-bottle pores declines sharply as the bottleneck of the ink-bottle pore is destroyed.^23^ The above situations make clear that the tectonic deformation weakens the particle size effect on the pore structures of deformed coal. The reason is that the tectonic deformations contribute to the fully developed pore structures in deformed coal.^1,17^ As for undeformed coal with an intact bedding plane, the pulverization process exerts a crucial influence on the pore structure, making the pore structure of undeformed coal gradually approach that of deformed coal in the same coal seam.

#### 3.1.2 Influence of the Particle Size on PV and SSA

**Figure 3:**
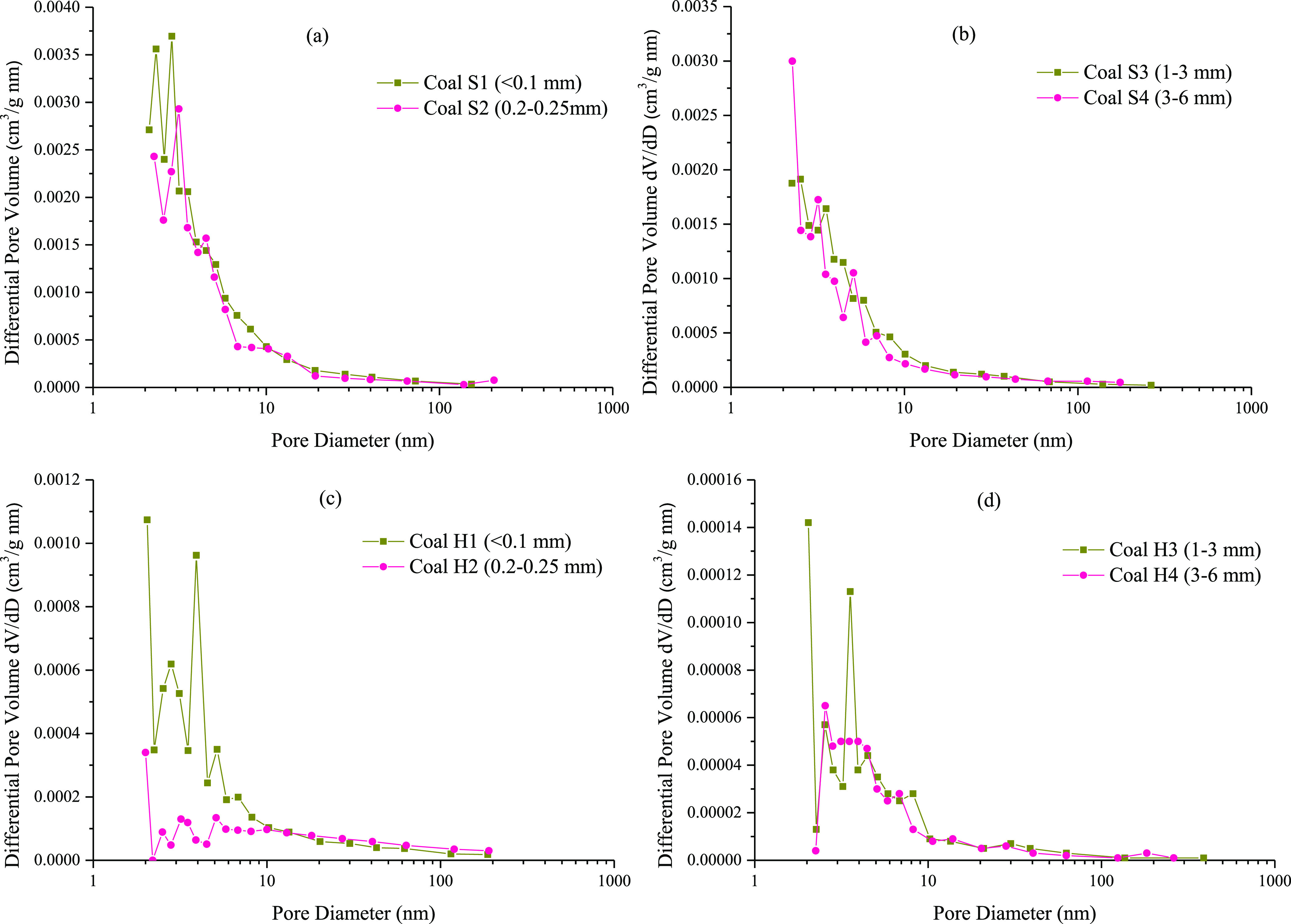
Distribution of the pore size of coal samples calculated using the BJH model. Note: (a) and (b) represent Coal S and (c) and (d) represent Coal H.

The gas adsorption capacity is greatly affected by the PSD. The BJH model was employed to calculate the PSD of Coal S and Coal H from the adsorption branch, and the results are shown in Figure 3. The larger the *d*V/*d*D value is, the greater the quantity of pores in the corresponding diameter becomes. The PSD feature of Coal S and Coal H with different particle sizes further validates the influence of the pulverization on the coal pore structure, especially the quantity of micropores. The tectonic deformation makes the pore structure of the deformed coal fully developed, and the mechanical pulverization can only transform its PSD by destroying some original pores. Undeformed coal has a positive response to mechanical pulverization. Under the action of mechanical pulverization, a great number of micropores and mesopores that were never present before will appear in the undeformed coal.

We adopted the BET, D-R, and BJH methods to quantitatively characterize the pore structure of coal and obtained the PV and SSA, as shown in Table 2. It should be noted that the BJH PV can be divided into mesopore and macropore volumes. The BJH PV and SSA of Coal S became larger with the decrease of the particle size. The BJH PV and SSA increased from 17.84 × 10^–3^ to 23.01 × 10^–3^ cm^3^/g and from 6.94 to 11.785 cm^2^/g, respectively. However, the micropore volume, micropore SSA, and BET SSA of Coal S showed a U-shape relationship with the particle size, while the minimum value occurs at the 1–3 mm particle size and the maximum value occurs at the 3–6 mm particle size. The variation of these parameters is different from the nitrogen adsorption amount, which contradicts the conventional viewpoint.^18^ The phenomenon occurs because the mesopore volume and SSA of Coal S1 (less than 0.1 mm) are nearly two times higher than those of Coal S4 (3–6 mm), and the difference in the micropore volume and SSA between Coal S1 and Coal S4 is rather small. Unlike Coal S, both the micropore volume and BJH PV as well as SSA (micropore, BET, and BJH) of Coal H showed a negative correlative with the particle size. The parameters of Coal H samples with a particle size larger than 1 mm have little variation, while there is a sudden increase when the particle is pulverized to a size less than 1 mm. What is noticeable is that the parameters of Coal H with the smallest particle size are 10 or 20 times larger than those with the largest particle size.

**Table 2:** Pore Structure Parameters from the N_2_ adsorption^a^

		PV (× 10^–3^ cm^3^/g)				SSA (m^2^/g)		
sample no.	particle size (mm)	micropore	mesopore	macropore	BJH PV	micropore	BJH SSA	BET SSA
S1	<0.1	10.215	15.898	7.122	23.020	28.748	11.785	26.268
S2	0.20 to 0.25	9.327	12.617	7.733	20.350	26.288	8.131	20.855
S3	1 to 3	9.030	11.034	8.448	19.482	25.451	7.695	18.975
S4	3 to 6	12.372	9.847	7.994	17.841	34.818	6.940	29.868
H1	<0.1	1.785	4.517	3.635	8.152	5.021	2.880	4.279
H2	0.20 to 0.25	0.903	3.326	4.386	7.712	2.594	1.165	1.751
H3	1 to 3	0.069	0.471	0.265	0.736	0.195	0.300	0.227
H4	3 to 6	0.061	0.411	0.335	0.746	0.171	0.247	0.201

a Note: the PV and SSA of the micropore were calculated by adopting the D-R method, while the PVs of meso- and macropores were calculated by adopting the BJH method.

**Figure 4:**
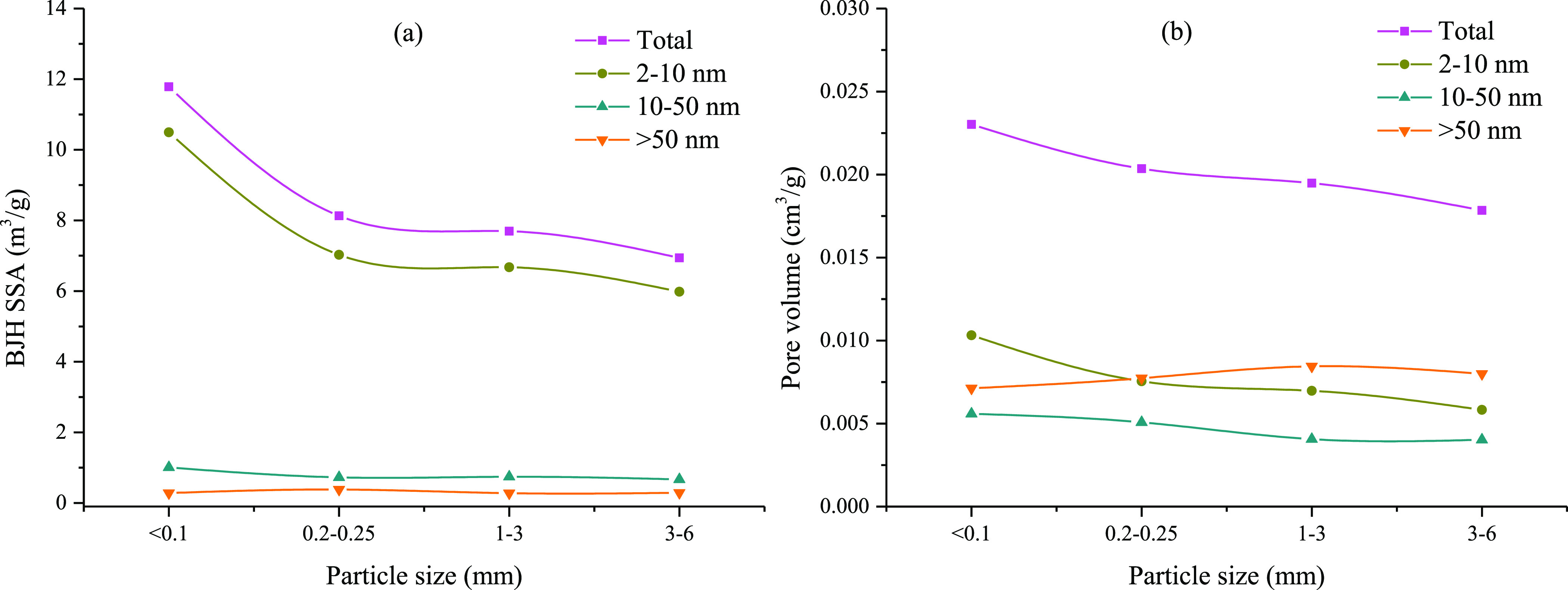
Pore structure parameters of meso- and macropores for Coal S. Note: (a) presents BJH SSA variation with the particle size and (b) presents PV variation with the particle size.

**Figure 5:**
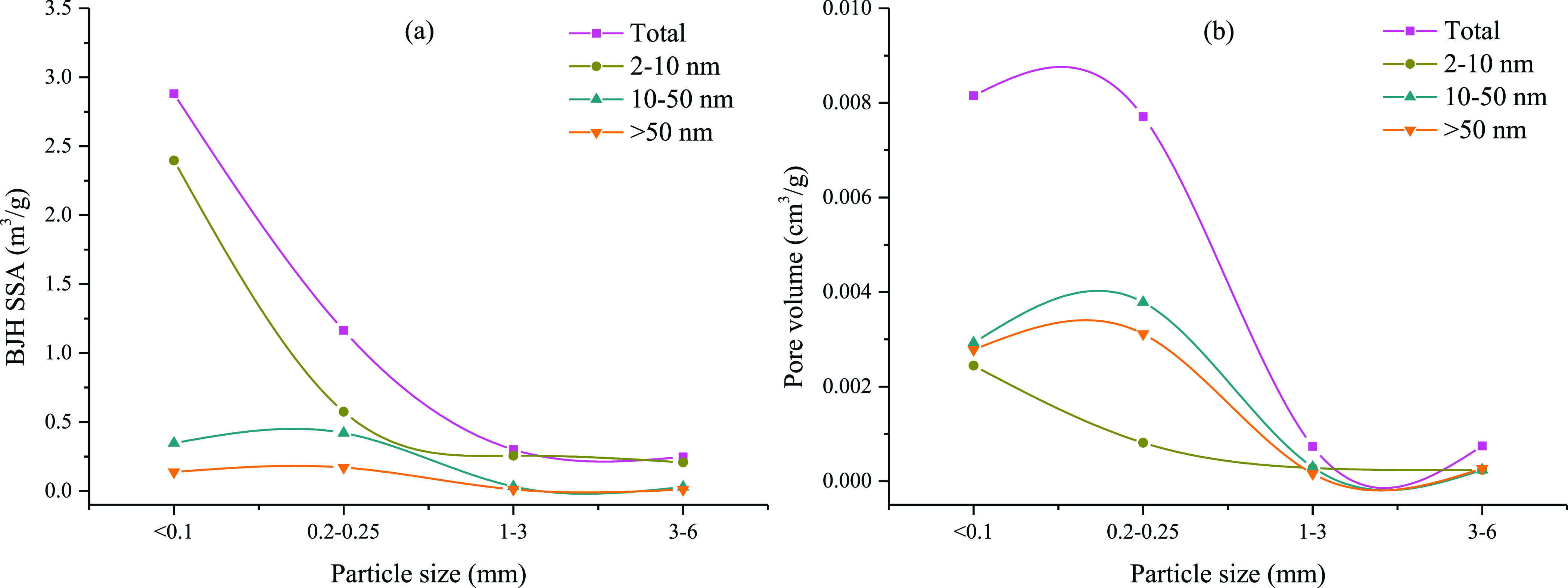
Pore structure parameters of meso- and macropores for Coal H. Note: (a) presents the BJH SSA variation with the particle size and (b) presents the PV variation with the particle size.

Pore structure parameters with different pore diameters of Coal S and Coal H and their variation with the particle size are shown in Figures 4, 5 and Table 2. Both PV and SSA of mesopores increase as the particle size gets smaller and smaller, which is consistent with previous observations.^16,17,31^ It should be noted that the fine mesopores (2–10 nm) dominate the variation of mesopores. Meanwhile, the large mesopores (10–50 nm) of Coal S show a slight change, as shown in Figure 4. However, for Coal H, there is no strictly positive correlation between the larger mesopores and the particle size, as shown in Figure 5. The crushing of the coal particle indeed exerts influence on the coal pore structure, but it imposes different effects on typical deformed and undeformed coal. For Coal H, more constricted openings exist in the larger particle size, but nitrogen molecules cannot enter the mesopore through the constricted entrance. The inaccessible mesopores to nitrogen molecules gradually become accessible by pulverizing. Moreover, it can be inferred from the SSA variation in Table 2 and Figure 5 that the diameter of such mesopores should be less than 10 nm. In addition, some mesopores with a diameter of 10–50 nm and macropores of sample S1 were destroyed. For Coal S, sample S4 has the maximum BET SSA because it retains more micropores formed in the period of tectonic deformation and coalification. When the particle size is smaller than that of S3, as the particle size decreases, the SSA of micropores gradually increases. However, the micropore SSA of Coal S1 is still smaller than that of Coal S4 (3–6 mm). However, the SSA of mesopores of Coal S1 is two times larger than that of Coal S4, which demonstrates that the pulverization of Coal S causes the micropores to connect with each other and become mesopores.

Compared to Coal H, the variation of the pore structure parameters of Coal S with the particle size is much smaller. Additionally, the constricted pores scarcely exist in Coal S, while such pores occupy a large proportion of Coal H. The reason is that the tectonic deformation strengthens the connectivity of the pore network and makes the original particle size smaller, which can weaken the effect of the particle size on the pore structure.^17^ Moreover, mechanical pulverization exerts much weaker influence on the coal particle compared with tectonic deformation, but crushing undeformed coal can also leak out the constricted pores formed in the coalification period.

### 3.2 Differences in Influence of the Particle Size on Fractal Characteristics of the Pore Structure between Deformed and Undeformed Coal

With a complicated pore-fracture structure and a rough pore surface, coal has the basis of gas adsorption and flow (desorption, diffusion, and seepage) law. It has been proved that the fractal geometry is an effective method to describe pore structures. Moreover, the adsorption capacity has been successfully analyzed using fractal dimensions.^32,33^ In this study, fractal dimensions are calculated by means of the FHH fractal model, which is based on low-temperature N_2_ adsorption experimental data.^32^ The equation is
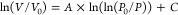
1

*A* can be obtained by plotting and fitting the gas adsorption isotherm data in light of ln*V* versus ln (ln (*P*_0_/*P*)). The slope of the fitting line should be equivalent to *A*. The fractal dimension *D* is dependent on *A*, and it has two different formulas. When *D* is used to analyze the structural characteristics of coal pores, *A* = *D*–3 is commonly applied. Therefore, we can calculate *D* using *D* = 3 + *A*. The details of the derivation have been described in previous research results.^34^

**Figure 6:**
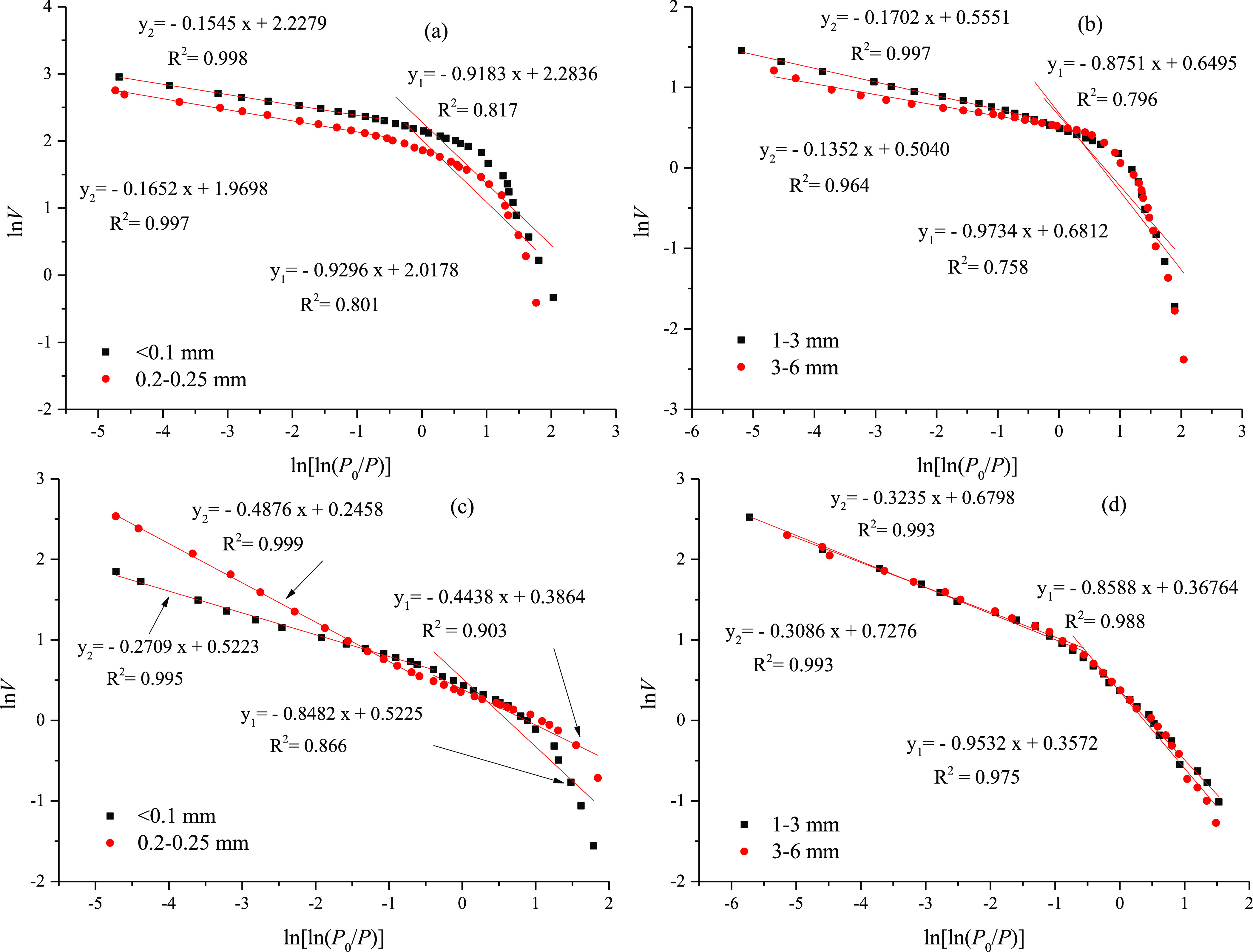
Plots of ln*V* vs ln[ln(*P*_0_/*P*)] reconstructed from the low-temperature N_2_ adsorption isotherms

**Note:** (a) and (b) represent Coal S, while (c) and (d) represent Coal H.

In the relatively low pressure regions (0 to 0.5), the gas adsorption mainly relies on the van der Waals force and micropore filling. However, in regions of relatively high pressures (0.5 to 1), the gas adsorption is mainly dependent on capillary condensation phenomena.^24^ The FHH plots of undeformed and deformed coal in this study are shown in Figure 6, and they have two obvious linear segments at the *P*/*P*_0_ intervals of 0 to 0.5 (Region 1) and 0.5 to 1 (Region 2) regions. The results of Region 2 all show a good fit. However, the fitting result of Region 1 is relatively weak.

The fractal dimensions are detailed in Table 3. On the whole, the difference in *D*_1_ values between Coal S and Coal H is small. As for Coal S, there is no apparent relationship between *D*_1_ and the decreasing particle size of deformed coal. From the isotherms, we can find that the micropore filling of deformed coal in the low pressure zone (*P*/*P*_0_ < 0.1) is particularly conspicuous and does not conform to the strict monolayer adsorption law. At this time, the adsorption result cannot adequately characterize the pore surface geometry, so the fractal scale law is not apparent; that is, the fractal dimension has a low fitting degree.^35^ Coal H with the particle sizes of 1–3 mm and 3–6 mm has a good fit in the low pressure zone, and *D*_1_ gets larger as the particle size gets smaller. However, the fitting degree of undeformed coal with particle sizes of 0.2–0.25 mm and smaller than 0.1 mm gradually decreases, which is related to the sudden increase of the PV of micropores. This phenomenon also agrees with the above section, Section 3.1.1, of N_2_ adsorption isotherm (*P*/*P*_0_ less than 0.1 mm). The proportion of micropores increases as the coal rank increases, while Coal H belongs to anthracite.^2^ The micropores in Coal S are fully leaked through long-term tectonic deformation, while Coal H still has many micropores that cannot be penetrated by nitrogen molecules. Therefore, Coal H will leak more micropores and fine mesopores as the particle size decreases, and these pores exacerbate its micropore filling.

**Table 3:** Fractal Dimension Calculations

		*P*/*P*_0_: 0 to 0.5			*P*/*P*_0_: 0.5 to 1		
sample No	particle size (mm)	*A*_1_	*D*_1_ = 3 + *A*_1_	*R*^2^	*A*_2_	*D*_2_ = 3 + *A*_2_	*R*^2^
S1	<0.1	–0.9183	2.0817	0.817	–0.1545	2.8455	0.998
S2	0.20–0.25	–0.9296	2.0704	0.801	–0.1652	2.8348	0.997
S3	1–3	–0.8751	2.1249	0.796	–0.1702	2.8298	0.997
S4	3–6	–0.9734	2.0266	0.758	–0.1352	2.8648	0.964
H1	<0.1	–0.8482	2.1518	0.866	–0.2709	2.7291	0.996
H2	0.20–0.25	–0.4438	2.5562	0.903	–0.4876	2.5124	0.999
H3	1–3	–0.8588	2.1412	0.988	–0.3235	2.6765	0.993
H4	3–6	–0.9533	2.0467	0.975	–0.3086	2.6914	0.993

The fractal dimension *D*_2_ and the particle size show a U-shaped correlation. The fractal dimension *D*_2_ reaches the minimum value at coal particle sizes of 1–3 and 0.2–0.25 mm of Coal S and Coal H, respectively. This may be caused by the variation of meso- and macropores and the increase of the PV with the decrease of particle size. As shown in Figure 4 and Figure 5, the increase of mesopores and macropores will reduce the nonuniformity of the PSD and lower the *D*_2_ accordingly. However, the PV will increase as coal particle size decreases. On the basis of fractal geometry theory, fractal dimension for PSD increases with the increase of porosity under the fixed range of pore size.^36^ Thus, the fractal dimension *D*_2_ can increase with a cumulative growth of the PV. As for Coal S and Coal H, when the particle size decreases from 3–6 mm to 1–3 mm and from 3–6 mm to 0.2–0.25 mm, respectively, the increase of mesopores dominates pore structures of coal, and the fractal dimension *D*_2_ decreases gradually. The influence of the PV will exceed that of pore numbers when the particle size further decreases. That is, *D*_2_ will get larger as the particle size decreases from 1–3 mm or 0.2–0.25 mm to smaller than 0.1 mm. Meanwhile, *D*_2_ of Coal H with a particle size smaller than 0.1 mm is closest to that of Coal S. Comparing and analyzing the two coal samples, we discover that *D*_2_ of Coal S is markedly larger than that of Coal H with the same particle size. This shows that the heterogeneity of the pore structure of Coal S is higher than that of Coal H. From this analysis, we know that the geological tectonic deformation intensifies the heterogeneity of the coal pore structure, making its original particle size smaller. That is why *D*_2_ variation of Coal S is less than that of Coal H with the decreasing particle size. The pulverization process of the coal particle makes the heterogeneity of the pore structure decrease first and then increase, but the critical value of the particle size of deformed and undeformed coal is different. The heterogeneity of the pore structure of Coal H with a particle size smaller than 0.1 mm is closest to that of Coal S, further indicating that the pore structure of Coal H approaches that of Coal S with the decreasing particle size.

### 3.3 Differences in Influence of the Particle Size on Gas Adsorption Capacity between Deformed and Undeformed Coal

**Figure 7:**
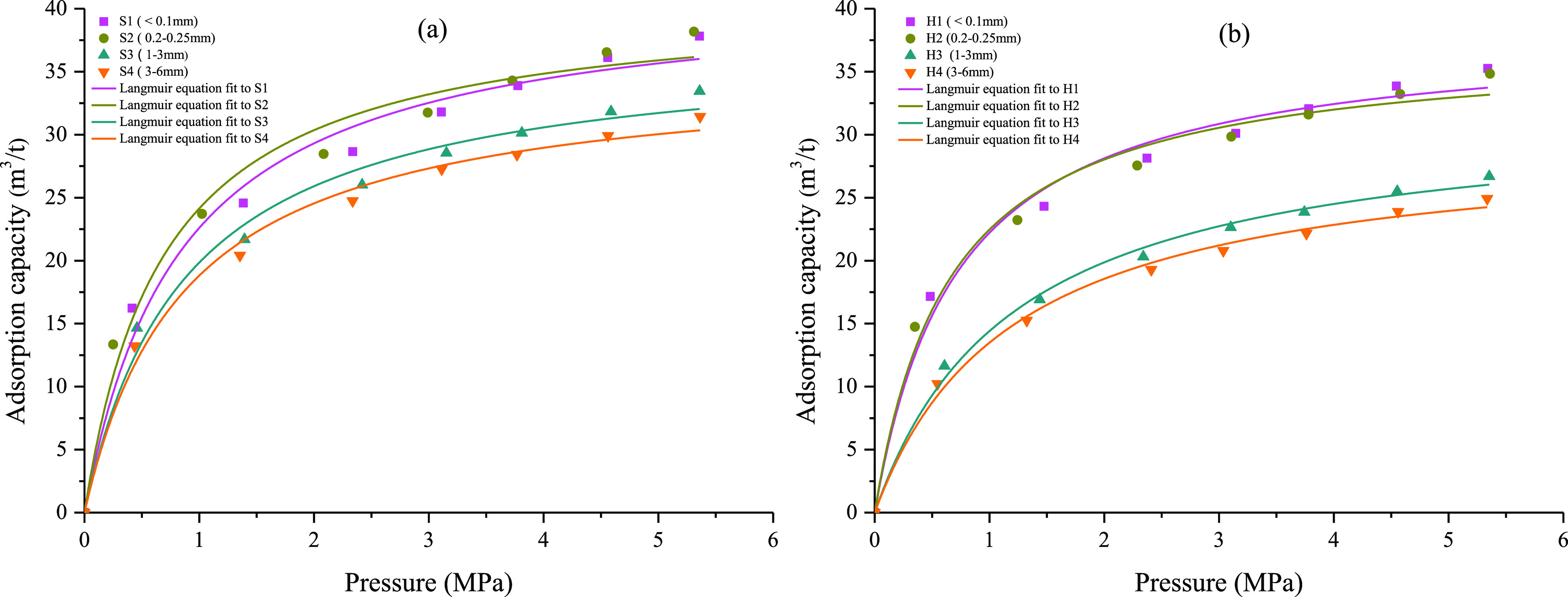
Adsorption rule of coal samples fitted by the Langmuir equation. Note: (a) represents Coal S and (b) represents Coal H.

**Figure 8:**
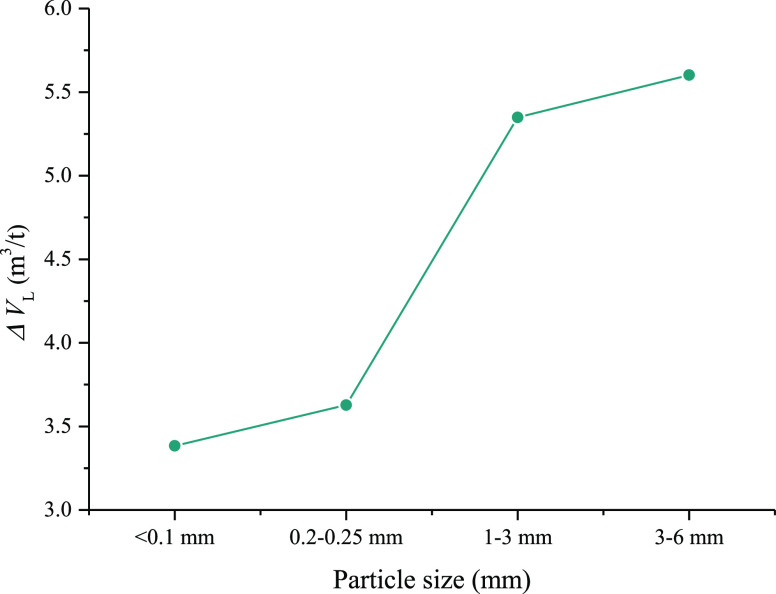
Langmuir volumes (*V*_L_) differences between Coal S and Coal H.

The methane adsorption experiments offer further support for analyzing the gas adsorption properties of coal. The methane adsorption rules of Coal S and Coal H fitted by the Langmuir equation are shown in Figure 7. The adsorption capacity of two kinds of coal samples both increases with the reduction of particle size, while that of Coal H increases even more. The result confirms the aforementioned variation rule of the pore structure and shows the control effect of the pore structure on the adsorption capacity. We listed the Langmuir parameters, which are calculated by statistically fitting the Langmuir model via nonlinear regression in Table 4. *V*_L_ represents the maximum adsorption volume of coal, and *P*_L_ correlates with the adsorption rate at the low-pressure stage. These two together affect the adsorption capacity of coal.^37,38^*V*_L_ of Coal S increases from 35.3079 to 41.6882 m^3^/t, and that of Coal H increases from 29.7059 to 38.3046 m^3^/t. One of the most remarkable things is that the former is larger than the latter at each particle size, which demonstrates that both the tectonic deformation and the mechanical pulverization increase the maximum adsorption volume of coal. There is no correlation between *P*_L_ of Coal S and the particle size, whereas the smaller the particle size of Coal H is, the smaller its *P*_L_ value is. These observations indicate that the gas adsorption capacity of the coal gradually increases with the decrease of the particle size. Meanwhile, the overall *V*_L_ difference (Δ*V_L_*) between Coal S and Coal H decreases with the decreasing particle size (Figure 8). The adsorption capacity of Coal H gradually approaches that of Coal S with the decreasing particle size. The above experimental results indicate that the adsorption capacity of coal is closely correlated with the degree of structural deformation and the size of the coal particle, which will be discussed in detail below.

**Table 4:** Analysis Results for Methane Adsorption Experiment

sample No.	particle size (mm)	langmuir volume (m^3^/t)	langmuir pressure (MPa)	*R*^2^
S1	<0.1	41.6882	0.8445	0.982
S2	0.20–0.25	40.8796	0.6932	0.982
S3	1–3	37.2977	0.8791	0.987
S4	3–6	35.3079	0.8766	0.992
H1	<0.1	38.3046	0.7243	0.988
H2	0.20–0.25	37.2518	0.6581	0.987
H3	1–3	31.9494	1.2167	0.995
H4	3–6	29.7056	1.2018	0.995

### 3.4 Discussion of Adsorption Differences

From the results and the discussion in Sections 3.1 and 3.2, it can be seen that the influence of tectonic deformation on the pore structure of coal is much larger than that of pulverization. The reason is that deformed coal belongs to the shear zone along with the coal seam, and it mainly undergoes ductile shear deformation. The strength and time of the tectonic compression and shearing action are much greater than the strength applied to the coal body during pulverization. However, Section 3.3 illustrates that the difference in adsorption capacity between deformed coal and undeformed coal is not as great as the difference in the pore structure. The adsorption capacity of the coal is jointly determined by various pore structure parameters.^23^ The gas adsorption mode in coal includes monolayer adsorption and micropore filling, capillary condensation occurring in mesopores and macropores, and multilayer adsorption occurring in macropores.^28^ The pulverization of coal particles can significantly subjoin the number of adsorption pores in undeformed coal, particularly micropores. It is a vital reason why the adsorption capacity of coal samples becomes larger as the particle size gets smaller (Table 2 and Figure 5). However, the variation of adsorption capacity with the particle size of deformed coal is less than that of undeformed coal because the tectonic deformation weakens the influence of the particle size. In contrast, the tectonic deformation significantly increases SSA of the coal and the proportion of mesopores and micropores. As a result, more adsorption sites can be provided for gas (Table 2). However, at the same time, by comparing the fractal dimension *D*_2_ of deformed coal and undeformed coal (Table 3), we find that the former has higher heterogeneity of the pore structure and higher capillary condensation on the pore surface, which results in the reduction of gas adsorption capacity.^24^ This is probably because the difference in the maximum adsorption volume between typical deformed and undeformed coal is not as significant as the difference in SSA (Table 2 and Table 4). Another reason is the adsorption layer thickness theory, meaning the amount of methane adsorption depends on the numbers of adsorbed layers in different pore sizes.^39^ Similarly, this is also the reason why the adsorption capacity of Coal S4 (3–6 mm) with a higher SSA is smaller than that of Coal S1 (less than 0.1 mm) with a smaller SSA.

For undeformed coal, the variation of *V*_L_ with the particle size demonstrates that the adsorption pore SSA, especially micropore SSA, dominates the adsorption capacity of coal, while the heterogeneity of the pore structure is a secondary factor. Due to the crushing effect, plenty of adsorption pores are generated. Therefore, the micropore SSA of the sample Coal H1 is 30 times larger than that of the sample Coal H4 (Table 2 and Figure 5). However, when the pore structure of coal is hugely developed, e.g., the typical deformed coal, the particle size exerts little influence on the adsorption pores (Table 2 and Figure 4). The heterogeneity of the pore structure is a significant factor affecting the adsorption capacity, while the adsorption SSA is a secondary factor. As the particle size gets smaller, undeformed coal with smaller particle size has more adsorption pores, and its heterogeneity of the pore structure is lower than that of deformed coal. This is the primary reason why the difference in adsorption capacity gradually decreases with the particle size.

## 4 Conclusions

To enhance the prediction accuracy of gas outburst hazard and CBM reserves, we experimentally studied the variation of the pore structure and adsorption capacity of deformed and undeformed coal with the particle size. The conclusions are as below:

(1) The adsorption-pore proportion in deformed and undeformed coal increases with the reduction of particle size. The influence of the particle size on deformed coal is to reshape the PSD of coal and connect some micropores into mesopores. The mechanical pulverization exerts positive influence on the pore structure of undeformed coal, especially the adsorption pores. Under applied mechanical stress, the number of ink bottle holes in the undeformed coal has a notable reduction, and the micropore filling is more pronounced. The pore structure of undeformed coal gradually approaches that of deformed coal with the decreasing particle size.

(2) The adsorption capacity differences between deformed and undeformed coal diminish with the decrease of the particle size. As the particle sizes decrease, the coal *V*_L_ value increases gradually, and the deformed coal *V*_L_ value is larger than that of undeformed coal at each particle size, while the coal *P*_L_ value decreases. The Δ*V_L_* between the undeformed and deformed coal is positively correlated with the particle size of the coal. The adsorption capacities of the undeformed coal had gradually approached those of the deformed coal with the decreases in the particle sizes, which is consistent with the variation law of pore structures with the particle size.

(3) In terms of *D*_1_ values, the FHH fractal model is useful to characterize the pore surface geometry of undeformed anthracite coal with a size greater than 1 mm but is inadequate to characterize the deformed coal and undeformed coal particle with a size less than 0.2–0.25 mm. It is proved that the applied stress has a certain influence on the small mesopores and micropores of undeformed coal. The relationship between the *D*_2_ value and coal particle size displays a U-shape curve. In addition, the minimum *D*_2_ value is at a particle size 0.2–0.25 mm. Due to the effect of applied stress, the *D*_2_ value of the undeformed coal is gradually close to that of deformed coal. This further indicates that as the particle size decreases, the pore structure of undeformed coal approaches that of deformed coal.

(4) The research on different rules and mechanisms in pore structures and gas adsorption capacities of deformed and undeformed coal detects that the adsorption capacity of coal becomes greater with the decreasing particle size, and the adsorption capacity differences between deformed and undeformed coal diminish with the decrease of the particle size. This study is significant for selecting the particle size of coal samples in predicting coal and gas outburst disasters and CBM productivity. Selecting particle size is an essential task for predicting CBM content and gas outburst disasters. Theoretically, according to the above adsorption law, we preferentially select coal samples with large adsorption capacity, that is, small particle size. Practically, the granularity is selected in terms of engineering operability. Therefore, the study is of great important guiding significance for further engineering practice.
